# Introducing ProsperNN—a Python package for forecasting with neural networks

**DOI:** 10.7717/peerj-cs.2481

**Published:** 2024-11-25

**Authors:** Nico Beck, Julia Schemm, Claudia Ehrig, Benedikt Sonnleitner, Ursula Neumann, Hans Georg Zimmermann

**Affiliations:** Fraunhofer IIS, Fraunhofer Institute for Integrated Circuits IIS, Nürnberg, Bavaria, Germany

**Keywords:** Price forecasting, Macroeconomic forecasting, Financial forecasting, Software, Recurrent neural networks

## Abstract

We present the package prosper_nn, that provides four neural network architectures dedicated to time series forecasting, implemented in PyTorch. In addition, prosper_nn contains the first sensitivity analysis suitable for recurrent neural networks (RNN) and a heatmap to visualize forecasting uncertainty, which was previously only available in Java. These models and methods have successfully been in use in industry for two decades and were used and referenced in several scientific publications. However, only now we make them publicly available on GitHub, allowing researchers and practitioners to benchmark and further develop them. The package is designed to make the models easily accessible, thereby enabling research and application in various fields like demand and macroeconomic forecasting.

## Introduction

Neural networks have become increasingly popular in many fields (Benidis et al., 2023), partly thanks to open source libraries like PyTorch (Paszke et al., 2019) and Tensorflow (Abadi et al., 2016). Building blocks such as long short term memories (LSTM) (Hochreiter & Schmidhuber, 1997) and gated recurrent units (GRU) (Cho et al., 2014) are central for the success of these frameworks because they allow engineers and scientists to share ideas in code easily and to effectively build on top of each other’s contributions (Benidis et al., 2023). The neural network architectures that are available within PyTorch and Tensorflow span from simple multi layer perceptrons (MLPs) to architectures designed for time series forecasting. Recurrent neural networks deal specifically with sequential data, and Elman recurrent units, GRUs, and LSTMs are popular implementations of these (Hewamalage, Bergmeir & Bandara, 2021). Nevertheless, Hewamalage, Bergmeir & Bandara (2021) find that these architectures do not substantially outperform ETS or ARIMA on four out of six evaluated datasets. Further, the time series community long held the belief that complex elaborate models are inferior to simple forecasting approaches, for example highlighted by the M3 competition, where no machine learning approach was under the top ranking participants (Makridakis & Hibon, 2000). This notion shifted with the M4 competition that was won by a hybrid model that uses exponential smoothing in combination with an LSTM neural network (Makridakis, Spiliotis & Assimakopoulos, 2018; Smyl, 2020). Similarly, the explainable version of N-Beats, that showed impressive performance on the M4 dataset, learns specifically seasonality and trend components (Oreshkin et al., 2019) and DeepAR, another state of the art time series forecasting neural network combines a recurrent neural network with a user-specified distributional assumption of the target variable (Salinas et al., 2020). Recently transformer-based architectures trained as time series foundation models achieve promising results (see for example Rasul et al., 2023).

The neural networks that are implemented in prosper_nn have been used successfully in industrial applications at Siemens over a decade before neural networks were widely used in time series forecasting (Zimmermann, Neuneier & Grothmann, 2000, 2001a, 2001b; Zimmermann et al., 2011, Zimmermann, Tietz & Grothmann, 2012; Zimmermann et al., 2012; Zimmermann, Grothmann & Tietz, 2012; Zimmermann, Tietz & Grothmann, 2013; Zimmermann, Grothmann & Von Mettenheim, 2013; Zimmermann, Grothmann & Tietz, 2016; Siekmann et al., 1999; Erdem & Zimmermann, 2001; Tokic et al., 2020). Since their implementations have not been published, only few members of the scientific community have been able to reproduce these results or evaluate the models (Von Mettenheim & Breitner, 2012; Von Mettenheim, 2014; Von Mettenheim & Breitner, 2014; Köpp, Von Mettenheim & Breitner, 2014; Hoffmann, 2015; Bjarnle & Holmström, 2015; Mvubu et al., 2020; Abdel-Karim, Benlian & Hinz, 2021; Rockefeller et al., 2022, 2023). With prosper_nn based on PyTorch, we provide the first open source implementation of Error Correction Neural Networks (ECNN) (Zimmermann et al., 2012), Historical Consistent Neural Networks (HCNN) (Zimmermann et al., 2012), Causal-Retro-Causal Neural Networks (CRCNN) (Zimmermann et al., 2012) and Fuzzy Neural Networks based on an Adaptive Neuro Fuzzy Inference System (ANFIS) approach, allowing experts to include prior knowledge (Siekmann et al., 1999). All these models were developed for time series forecasting problems and achieve promising results (Zimmermann et al., 2012; Zimmermann, Grothmann & Von Mettenheim, 2013; Zimmermann, Tietz & Grothmann, 2013; Rockefeller et al., 2022, 2023). ECNNs are a further development of RNNs (Rumelhart, Hinton & Williams, 1986), using prediction errors to improve the hidden state. HCNNs and CRCNNs also make use of the prediction errors, but model multivariate data instead of using external input. Whereas HCNNs can only model dynamics going in one direction, CRCNNs use two HCNNs internally so that backward information is added, similar to a bidirectional RNN (Schuster & Paliwal, 1997). Lastly, fuzzy neural networks allow creating interpretable neural networks by imposing an interpretable structure on a multilayer perceptron (MLP). We implement the mentioned models together with two tools for model evaluation. The uncertainty heatmap illustrates the scattering and accumulation of the individual forecasts in an ensemble. The sensitivity analysis shows the gradient of model predictions with respect to the model input for the use of interpretation and feature selection. These tools can be applied to other PyTorch models as well.

The implemented models differ from currently widely used neural networks in the following aspects: (i) Many of the common architectures use LSTMs as one central building block (for example Smyl, 2020; Salinas et al., 2020). The implemented ECNN, HCNN and CRCNN cells might offer an alternative, as illustrated by their success in applications and our benchmark. (ii) They are throughout free of distributional assumption (for example in contrast to DeepAR). (iii) They do not rely on common statistical time series patterns (in contrast to N-Beats or the model by Smyl (2020)). Table 1 summarizes the addressed modeling challenges by the different neural networks in prosper_nn.

**Table 1:** Summary of addressed modeling challenges and solutions.

	Modelling challenges	Solution	Publications
ECNN	Initial state is equal for all inputs and therefore does not represent the related model state for a given input. In each period new errors are included into the hidden state by model and data errors.	Teacher forcing.	Erdem & Zimmermann (2001), Mvubu et al. (2020), Zimmermann, Neuneier & Grothmann (2000, 2001a, 2001b), Zimmermann, Tietz & Grothmann (2012), Zimmermann et al. (2012)
HCNN	Unknown exogenous input variables in the future. Limited data availability.	Predicting all variables together (nonlinear adaption of the Vector autoregressive model). Efficient data usage by giving perfect feedback to the model. Including the known data in the state.	Abdel-Karim, Benlian & Hinz (2021), Bjarnle & Holmström (2015), Hoffmann (2015), Köpp, Von Mettenheim & Breitner (2014), Von Mettenheim (2014), Von Mettenheim & Breitner (2012, 2014), Rockefeller et al. (2022), Tokic et al. (2020), Zimmermann, Grothmann & Von Mettenheim (2013), Zimmermann et al. (2011), Zimmermann, Grothmann & Tietz (2016), Zimmermann, Tietz & Grothmann (2012),Zimmermann, Grothmann & Von Mettenheim (2013), Zimmermann et al. (2012)
CRCNN	Effects with a backward direction in times influence the forecasted variable.	Employ two HCNNs, one in forward and one in backwards time direction.	Zimmermann, Grothmann & Tietz (2012), Zimmermann, Tietz & Grothmann (2012), Zimmermann et al. (2012)
ANFIS	Black-box predictions that are hard to interpret.	Impose an interpretable rule-based structure on an MLP.	Siekmann et al. (1999)

In this article we explain only as much theory of the models as is necessary to understand the implementations. For a deeper theoretical understanding, we refer to the related articles. Additionally, we only mention the most important parameters in the code sections, but more options are available for users to modify. The presented package provides ready to use models, along with extensive documentation in the form of API references and detailed tutorials for all models and utilities. The modular implementation makes it easy for users to further develop the models and adapt the code.

Next we present the models and methods implemented in the prosper_nn package. For each, we give a short motivation, followed by the essential theory and a description of our implementation. This is followed by an illustrative case study and a discussion.

## Software implementation

As a framework in which we describe the models, we define a multivariate multistep forecast problem with exogenous variables. Let ${\bf y}_{t}\in{\mathbb R}^{n}$ for $t = 1, \ldots ,T$ be an $n$-dimensional target time series of length *T*. We are interested in forecasting the next $\tau$ values of ${{\bf{y}}_t}$ for the periods $t = T + 1, \ldots ,T + \tau$. The forecast for ${{\bf{y}}_t}$ is denoted as ${\widehat {\bf{y}}_t}$. Additionally, we have $m$ exogenous variables ${\bf{x}}_t \in {\mathbb {R}}^{m}$ for $t = 1, \ldots ,T + \tau$.

### Error correction neural network

Like LSTM and GRU, the ECNN architecture (Zimmermann, Neuneier & Grothmann, 2001b) is based on RNNs. All three treat exogenous variables as model input and use the hidden state of the previous time step for calculating a new state. Although the initial state is learned during training and represents the best fit for the training data, it is equal for all inputs and therefore does not represent the related model state for a given input. For RNNs like GRU or LSTM this misspecification is forwarded without any correction and is still present in the prediction. For long input time series the effect of the initial state will vanish, but for short time series it can deteriorate forecast accuracy. Additionally, in each period new errors are included into the hidden state by model and data errors. While LSTMs and GRUs employ gates to enhance the hidden state, ECNNs use teacher forcing, *i.e*., calculate the prediction error in past time steps ( $t \le T$) and hand it to the respective next hidden state (Williams & Zipser, 1989). By doing this, the error which was present in the hidden state can be mitigated for the next time step, since there is correcting feedback from the data. This can improve predictions for short-term forecasting. Note, that teacher forcing in most literature hands over the target value directly to the next hidden state, *e.g*., Goodfellow, Courville & Bengio (2016, p.377f), whereas ECNNs subtract the prediction first, thereby passing the prediction error. Further explanations of ECNNs can be found in literature, see for example Zimmermann, Neuneier & Grothmann (2001a) or Zimmermann, Tietz & Grothmann (2012, p. 693f).

ECNNs are similar to echo state networks (Jaeger, 2001), in that they are both based on RNNs. In contrast to ECNNs, echo state networks provide additional connections from the input to the output nodes, connections between the output nodes, possibly different non-linear functions for the hidden and output nodes, and do not distinguish between past and future time steps. The training of the networks also differs: echo state networks use conventional teacher forcing and only train the weights between the hidden state and the output, since the echo state property of long-term memory only depends on the weights between the hidden states. While ECNNs rely on the error backpropagation algorithm (Rumelhart, Hinton & Williams, 1986) for training, echo state networks can be trained by solving a linear regression task, *e.g*., by ordinary least squares.

The idea of teacher forcing in ECNNs is similar to FORCE learning (Sussillo & Abbott, 2009), which also aims to adjust the network weights such that the error is as small as possible after training and hence external input becomes redundant. While ECNNs use the errors as additional input and learn how to use it to correct the hidden state, FORCE learning provides the hidden states with the estimation of the target without training these connections. A major difference between the two teacher forcing algorithms is that FORCE learning aims for a massive weight update after the first epoch and keeping updates small afterwards, while teacher forcing in ECNN is updated as other weights in the network. The training of the weights between the hidden states in ECNNs corresponds to innate model training for echo state networks (Laje & Buonomano, 2013).

Hence, the architecture and training of ECNNs is similar, but not the same, to combining FORCE learning on echo state networks with innate training of the weights between the hidden states.

**Figure 1:**
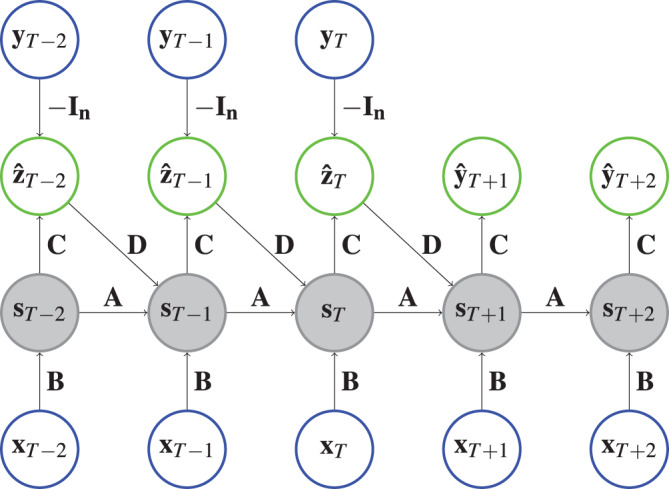
Architecture of an ECNN. Each circle represents a vector and each arrow depicts a matrix vector multiplication of the vector at the arrow origin with the matrix on the arrow. The state vectors are shaded to indicate that a hyperbolic tangent is applied. For example, $\hat{\bf z}_{t}={\bf C}\; {\rm tanh}\;({\rm s}_t)-{\bf I}_{n}{\bf y}_{t}$, where ${\bf I}_{n}$ represents the $n \times n$ identity matrix. Input vectors are in a blue circle and output vectors are shown in a green circle.

**Theory. **We focus on the use of the prediction error in the ECNN since the rest of the architecture is equivalent to a basic RNN. To calculate the prediction error, we subtract the known observation from the model output: ${\widehat {\bf{z}}_t} = {\widehat {\bf{y}}_t} - {{\bf{y}}_t}$. Afterwards, the error is multiplied with a matrix ${\bf D}\in {\mathbb R}^{k \times n}$ and added to the calculation of the next state. During training, the matrix ${\bf{D}}$ learns how to best incorporate the prediction error of the present period to improve the predictions of the following periods. The underlying assumption is that it is possible to learn where the cause of the error in the previous state stems from and that it is possible to mitigate it for the states of the following periods. So that the error $\varepsilon_{t}$ in the state ${s_t}$ that causes the deviation from the prediction to the target, is reduced by the teacher forcing: $\varepsilon_{t}\ge\varepsilon_{t}+{\bf D}\hat{\bf z}_{t}$. As we can only calculate the error if the actual data is available, this is only possible for past values ( $t \le T$). For the state transition and prediction equations follows:

(1) $$\eqalign{ {{\bf{s}}_{t + 1}} & = \left\{ {\matrix{ {tanh({\bf{A}}{{\bf{s}}_t} + {\bf{B}}{{\bf{x}}_{t + 1}} + {\bf{D}}({{\widehat {\bf{y}}}_t} - {{\bf{y}}_t}))} \hfill & {t \le T} \hfill \cr {tanh({\bf{A}}{{\bf{s}}_t} + {\bf{B}}{{\bf{x}}_{t + 1}})} \hfill & {t \;> \; T}  \cr } } \right.\cr {\hat {\bf{z}}_t} & = {\bf{C}}\tanh ({{\bf{s}}_t}) - {{\bf{y}}_t} \qquad \qquad \qquad \qquad \quad \;\;  t \le T\\ \hat {\bf{y}}_t & = {\bf{C}}\tanh ({{\bf{s}}_t}) \qquad \qquad \qquad \qquad \qquad \quad \;\;\; t \; > \; T}$$

where ${{\bf{s}}_t} \in {\mathbb R}^{k}$ is the state vector, ${\widehat {\bf{z}}_t}$ the prediction error and ${\bf{A}} \in {\mathbb R}^{k \times k}$, ${\bf{B}} \in {\mathbb R}^{k \times m}$ and ${\bf{C}} \in {\mathbb R}^{n \times k}$ matrices. These relations can be seen graphically in Fig. 1. During training, we minimize ${\widehat {\bf{z}}_t}$. Without data to correct the state (*e.g*., in the future), the hidden state can deteriorate over the horizon in case the model relies too much on the correction mechanism. Hence, ECNNs are most suited for short-term forecasting.

**Implementation. **Like the PyTorch implementation of RNNs, we implement cells that model one time step. The ECNN is stacked together by chaining multiple ECNN cells. For ECNN, the cells are designed such that they get the hidden state ${{\bf{s}}_{t - 1}}$ and observation ${{\bf{y}}_{t - 1}}$ of the last time step and the input ${{\bf{x}}_t}$ of the present time step. The cell then calculates the model error ${\widehat {\bf{z}}_{t - 1}}$. The teacher forcing is employed by first applying a torch.nn.Linear layer to transform ${\widehat {\bf{z}}_{t - 1}}$ and afterwards adding it to the new state at time $t$, ${{\bf{s}}_t}$. Finally, the cell returns the model error and the new model state ${{\bf{s}}_t}$. The implementation allows cases where ${{\bf{x}}_t}$ is available for $t = 1, \ldots ,T + \tau$ or $t = 1, \ldots ,T$ (see ‘Case Study’ page 18) by switching between future_U = True and future_U = False respectively. The whole ECNN (as depicted in Fig. 1) can be initialized with the parameters n_features_U (the dimension of the exogenous variables $m$), n_state_neurons (defining the size of the hidden state $k$), past_horizon (setting the look back of the model) and forecast_horizon (setting the number of steps to predict into the future):


ecnn = ECNN(n_features_U, n_state_neurons, past_horizon,
    forecast_horizon, future_U=True)

The class ECNN is a torch.nn.Module that can be used and trained in the same way as any other module in PyTorch. To train in mini-batches of size batchsize, we pass suitable data ${\rm x} \in {\mathbb R}^{({\tt past\_horizon} + \tau ) \times {\tt batchsize} \times m}$ and Y $\in {\mathbb R}^{{\tt past\_horizon} \times {\tt batchsize} \times n}$ to the model. It will return a torch.Tensor of the shape (past_horizon + forecast_horizon, batchsize, n) with the absolute errors in the first past_horizon entries of the first dimension and the prediction in the last forecast_horizon entries:


ecnn_output = ecnn(X, Y)
absolute_error = ecnn_output[:past_horizon]
forecast = ecnn_output[past_horizon:]

### Historical consistent neural network

Most RNNs assume that the exogenous input variables are known in the past and in the future. This is often not fulfilled. Vector-auto-regressive models (VAR) (Stock & Watson, 2001) address this deficiency. They use multivariate input data and learn the linear dependencies between all time series to predict the future of all series. One non-linear adaption of this idea in the neural network context is the HCNN based on Zimmermann et al. (2011), which produces accurate forecasts in various practical use cases like wind forecasting (Rockefeller et al., 2023) or predicting commodity prices for procurement (Zimmermann, Grothmann & Von Mettenheim, 2013). As is common in most of these use cases, we consider the multivariate target time series ${y_t}$ with no input ${x_t}$. HCNNs can be considered as ECNNs with a special structure for multivariate forecasting. For example, it reduces parameters by fixing the teacher forcing connection ${\bf{D}}$ and is able to learn a representation of ${{\bf{y}}_t}$ in the upper part of ${{\bf{s}}_t}$.

**Theory. **Again, the output consists of the prediction errors along the past horizon and the forecasts of ${{\bf{y}}_{\bf{t}}}$ for the future. But this time, the matrix between state and prediction is fixed and just extracts the first part of the hidden state:

(2) $$\eqalign{{\hat {\bf{z}}_t} & = [{{\bf{I}}_n},{{\bf{0}}_{n,k - n}}] \cdot {{\bf{s}}_t} - {{\bf{y}}_t}\quad{t \le T}\\{\hat {\bf{y}}_t} & = [{{\bf{I}}_n},{{\bf{0}}_{n,k - n}}] \cdot {{\bf{s}}_t}\quad\quad\quad{t \; > \; T}}$$

where ${{\bf{s}}_t} \in {\mathbb R}^{k}$ is the state vector with $k \ge n$, ${{\bf{I}}_n}$ an $n \times n$ identity matrix, ${{\bf{0}}_{n,k - n}}$ an $n \times k - n$ zero matrix and $[ \cdot , \cdot ]$ the column-wise concatenation of two matrices so that $[{{\bf{I}}_n},{{\bf{0}}_{n,k - n}}] \in {{\mathbb R}^{n \times k}}$.

Again, we have two cases for the state transition (see Eq. (3)), depending on whether the prediction error is used to correct the hidden state. A state ${{\bf{s}}_{t + 1}}$ for $t \le T$ is calculated from the previous state ${{\bf{s}}_t}$ corrected by the prediction error ${\widehat {\bf{z}}_t}$.

**Figure 2:**
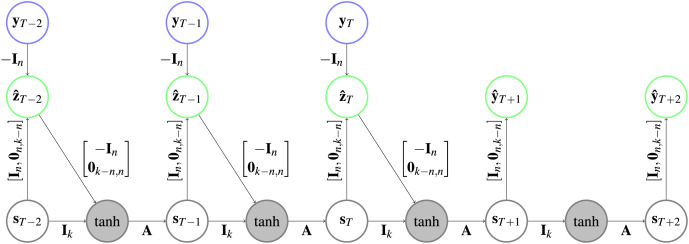
Architecture of an HCNN. In the gray circles a $\tanh$ is applied after the two vectors are added. Further explanation about the type of representation can be found in Fig. 1.

In the future ( $t \; > \; T$), the state follows only from the previous state (Eq. (3)). In both cases a state transition matrix ${\bf{A}} \in {\mathbb R}^{k \times k}$ is applied afterwards. Figure 2 depicts an HCNN architecture.

(3) $${{\bf{s}}_{t + 1}} = \left\{ {\matrix{ {{\bf{A}}\;tanh({{\bf{s}}_t} - {{[{{\bf{I}}_n},{{\bf{0}}_{n,k - n}}]}^ \top } \cdot {{\widehat {\bf{z}}}_t})} \hfill & {t \le T} \hfill \cr {{\bf{A}}\;tanh({{\bf{s}}_t})} \hfill & {t \;> \; T} \hfill \cr } } \right.$$

Unlike with the ECNN, the error correction here injects the true values like in common teacher forcing literature. Therefore, the intermediate state ${\tilde {\bf s}_t}$ contains ${{\bf{y}}_t}$ in the first $n$ entries before the hyperbolic tangent is applied, as seen in the following calculation.

(4) $${\tilde {\bf s}_t} = {{\bf{s}}_t} - {[{{\bf{I}}_n},{{\bf{0}}_{n,k - n}}]^ \top } \cdot {\widehat {\bf{z}}_t}$$

(5) $$\;\;\,\, = {{\bf{s}}_t} - {[{{\bf{I}}_n},{{\bf{0}}_{n,k - n}}]^ \top } \cdot ({\bf{s}}_t^{[1, \ldots ,n]} - {{\bf{y}}_t})$$

(6) $$ \;\;\,\, = {{\bf{s}}_t} - {[({\bf{s}}_t^{[1, \ldots ,n]} - {{\bf{y}}_t}),{{\bf{0}}_{k - n}}]^ \top }$$

(7) $$ \;\;\,\, = {[{{\bf{y}}_t},{\bf{s}}_t^{[n + 1, \ldots ,k]}]^ \top },$$

where $s_t^{[1, \ldots ,n]}$ is the state vector at time $t$ with only the first $n$ entries.

**Implementation.** HCNNs are implemented using cells that model one time step. The cell calculates the prediction and the next state according to Eqs. (2) and (3). If ${\bf{y}}$ is given to the cell, equations for $t \le T$ are used. Otherwise, equations for $t \; > \; T$ are applied.

The matrices $[{{\bf{I}}_n},{{\bf{0}}_{n,k - n}}]$ and ${[{{\bf{I}}_n},{{\bf{0}}_{n,k - n}}]^ \top }$ do not require gradients in the implementation, which keeps them unadjusted during training. However, they are registered as torch.nn.Parameter’s so that they are also moved to GPU if the model is moved to GPU. The whole HCNN is initialized with n_state_neurons (the number of state neurons $k$), n_features_Y (the number of variables to be predicted $n$), the past_horizon and the forecast_horizon.


hcnn = HCNN(n_state_neurons, n_features_Y, past_horizon,
    forecast_horizon)

The output of the HCNN has the shape (past_horizon + forecast_horizon, batchsize, n) and contains the in-sample absolute prediction errors in the first past_horizon entries of the first dimension and the forecasts in the remaining entries.


hcnn_output = hcnn(Y)
absolute_error = hcnn_output[:past_horizon]
forecast = hcnn_output[past_horizon:]

### Causal-retro-causal neural network

CRCNNs are bidirectional HCNNs proposed in Zimmermann, Grothmann & Tietz (2012). The term of causality refers to the characteristic of HCNNs to model outputs as a function of hidden states which only contain information up to the output time. This can be seen as a non-linear interpretation of Granger causality (Granger, 1969). Like bidirectional RNNs (Schuster & Paliwal, 1997), CRCNNs are meant to model both information going forward in time (causal) and information going backward in time (retro-causal). This can be an advantage in macroeconomic forecasting as expectations of future values often influence present decisions, for instance, the expectation of high future share values leading to an increase in stock purchases and therefore a rise in current share values (Veronesi, 2000).

**Figure 3:**
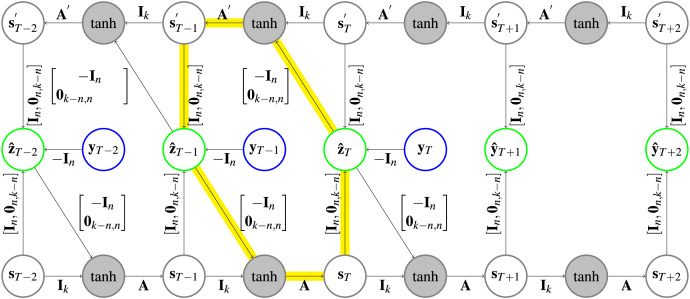
Architecture of a CRCNN. The yellow arrows highlight one of the circles that is present in the architecture. In the gray circles a $\tanh$ is applied after the two vectors are added. Further explanation about the type of representation can be found in Fig. 1.

**Theory.** A basic CRCNN consists of one HCNN model going forward in time and one HCNN model going backward. Accordingly, the state ${\bf{s}}$ keeps the information that are necessary for ${\bf{A}}$ to reproduce the dynamics in the forward time direction on the one hand. On the other hand, ${\bf{s}}^\prime$ and ${\bf{A}}^\prime$ reproduce the dynamics in the backward time direction. The prediction value for the target at time $t$ is the sum of the prediction values, which combines the causal and the retro-causal HCNN. The two HCNNs are only connected *via* the shared targets ${\widehat {\bf{z}}_t}$ (see Fig. 3). Accordingly, the state transition and prediction equations are:

(8) $$\eqalign{{{\bf{s}}_{{\bf{t}} + {\bf{1}}}} & = \left\{ {\matrix{ {{\bf{A}}\tanh ({{\bf{s}}_t} - {{[{{\bf{I}}_n},{{\bf{0}}_{n,k - n}}]}^ \top } \cdot {{\widehat {\bf{z}}}_t})} \hfill & {t \le T} \hfill \cr {{\bf{A}}\tanh ({{\bf{s}}_t})} \hfill & {t\; \gt\; T} \hfill \cr} } \right.\\{\bf{s}}{^\prime _{{\bf{t}} - {\bf{1}}}} & = \left\{ {\matrix{ {{\bf{A}}^\prime \tanh ({\bf{s}}{^\prime _t} - {{[{{\bf{I}}_n},{{\bf{0}}_{n,k - n}}]}^ \top } \cdot {{\hat {\bf{z}}}_t})} \hfill & {t \le T} \hfill \cr {{\bf{A}}^\prime \tanh ({\bf{s}}{^\prime _t})} \hfill & {t\; \gt\; T} \hfill \cr } } \right.\\{\hat {\bf{z}}_t} & = [{{\bf{I}}_n},{{\bf{0}}_{n,k - n}}]({{\bf{s}}_t} + {\bf{s}}{^\prime _t}) - {{\bf{y}}_t}\quad\quad\qquad t \le T\\{\hat {\bf{y}}_t} & = [{{\bf{I}}_n},{{\bf{0}}_{n,k - n}}]({{\bf{s}}_t} + {\bf{s}}{^\prime _t})\qquad\qquad\qquad t\; \gt\; T.}$$

Parameter $k \ge n$ is the dimension of both of the forward ${\bf{s}}$ and backward ${\bf s}^\prime$ state vectors.

**Figure 4:**
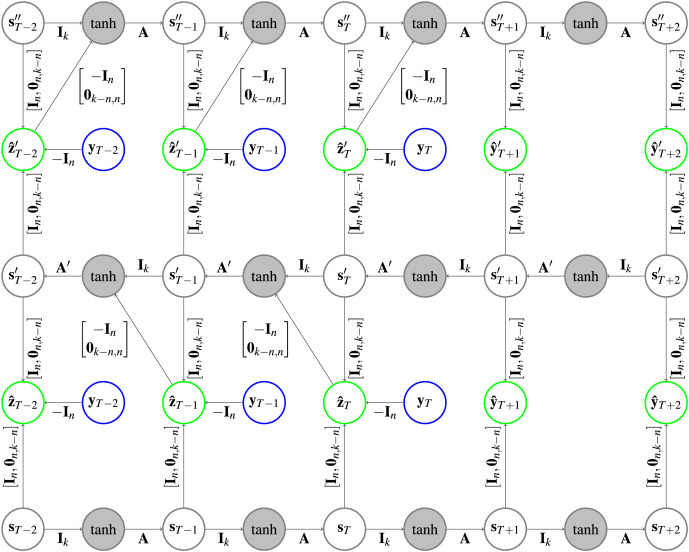
Architecture of a CRCNN as implemented in the software. No loops are present in the architecture anymore, which allows the error backpropagation algorithm to converge. This is achieved by alternatingly stacking HCNNs in forward and backward time direction, where we drop the error correction in the bottom HCNN. In the gray circles a $\tanh$ is applied after the vectors are added. Further explanation about the type of representation can be found in Fig. 1.

**Implementation. **The use of the prediction error to correct the hidden states for both HCNNs will lead to loops (following the arrows on the highlighted path in Fig. 3 leads back to the start) in the CRCNN architecture and the error backpropagation algorithm will not converge. This is why we unfold the loop by adding copies of the HCNNs to the CRCNN model. We proceed sequentially, so that each prediction is the sum of predictions of two successive models. The prediction error is then only used for the second of every successive pair of HCNNs, which eliminates the loops while still using the prediction error on every branch but the first one. This is depicted in Fig. 4 with the smallest possible version of three branches. Teacher forcing is not applied to the bottom branch. However, for the remaining branches it is applied from one target either to the causal or the retro-causal branch, but never both. We call these HCNN models branches of the CRCNN model, with causal and retro-causal branches alternating. All causal branches share the same matrix weights as do all retro-causal branches. The total number of causal and retro-causal branches is given by n_branches.

As a retro-causal HCNN can be achieved by using HCNN cells and passing the data in reverse direction to a generic (causal) HCNN, no extra implementation of a CRCNN cell is necessary. Because of the weight sharing, we only need two HCNN cells to build a CRCNN.

A CRCNN can be initialized in the following way:


crcnn = CRCNN(n_state_neurons, n_features_Y, past_horizon,
    forecast_horizon, n_branches)

The extra parameter compared to an HCNN is defined as n_branches, which sets the total number of branches of the CRCNN.

Training is very similar to that of a single HCNN. The only difference is that the CRCNN output contains errors and forecasts for every pair of causal and retro-causal HCNNs in the model (so the first dimension contains n_branches − 1 entries).

For forecasting, we take the output of the last causal branch as it has profited most from the correction with the prediction error.


crcnn_output = crcnn(Y)
absolute_errors = crcnn_output[:, :past_horizon]
forecast = crcnn_output[−1, past_horizon:]

### Fuzzy neural network

**Figure 5:**
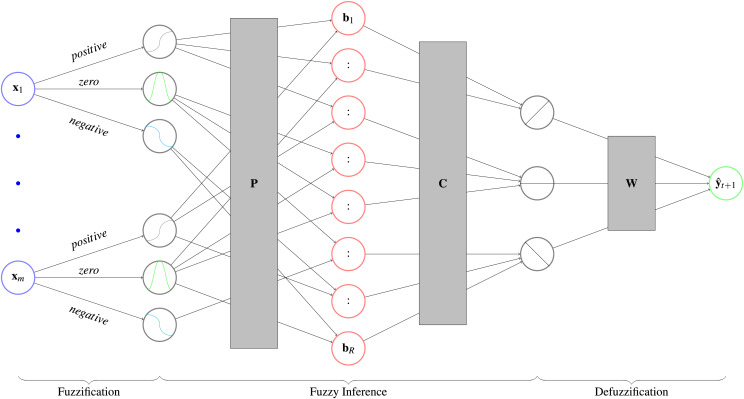
Architecture of a fuzzy neural network with its three parts. First, the *fuzzification* determines to which degree a variable ${x_i}$ is negative, zero, or positive. Second, an interpretable prediction is calculated by *fuzzy inference*, which applies the user defined rules ${\bf{P}}$ to get the degree of fulfillment ${\bf{b}}$. Afterwards, it calculats the respective consequences ${\bf{C}}$ that show to which degree the prediction is negative, zero or positive. In the third step, the *defuzzification*, the prediction is converted back with matrix ${\bf{W}}$ into a numerical prediction.

MLPs are considered black box predictors (Beck, 2018). Fuzzy neural networks whiten this black box by imposing an interpretable structure on an MLP. To make the numerical input interpretable, it classifies it as negative, zero or positive. This step is called *fuzzification*. The user can then propose rules for the network to follow, which are encoded in the network weights (*fuzzy inference*). This creates an interpretable output like negative, zero or positive that is transformed back to a numerical prediction (*defuzzification*). For an overview see Fig. 5. These types of fuzzy neural networks are known as ANFIS models. Walia, Singh & Sharma (2015) published a survey about the models, where they give an overview over previous research. Furthermore, implementations in PyTorch are provided by Lenhard & Maringer (2022) and https://github.com/jfpower/anfis-pytorch. In contrast to these packages, our implementation is based on Siekmann et al. (1999), which allows experts to include their prior knowledge in the form of rules into the *fuzzy inference* step. With these rules, experts are able to set relationships between exogenous variables and the target variable beforehand. During training the model weights the output of the rules, which can be seen as a degree of belief in each rule. Afterwards, experts can evaluate the rules by comparing in which rules they believed and in which the model does. We implemented the model for classification and regression, but focus on the later case in the following.

**Theory. **The first step of the fuzzy neural network is *fuzzification* which translates numerical input into a verbal description. This is achieved with the help of three so called membership functions, consisting of two logistic function and a Gaussian function:

(9) $$\eqalign{m{f_0}(x) & = m{f_{neg\_normlog}}(x) = {1 \over {1 + \exp ( - 4(\lambda x - 0.5))}}{\mathrm{ with }}\;\lambda \;< \;0 \\ m{f_1}(x) & = m{f_{gauss}}(x) = \exp \left( { - {1 \over 2}{{\left( {{x \over \sigma }} \right)}^2}} \right)\\ m{f_2}(x) & = m{f_{normlog}}(x) = {1 \over {1 + \exp ( - 4(\lambda x - 0.5))}}{\mathrm{ with }}\;\lambda\; \gt\; 0.}$$

**Figure 6:**
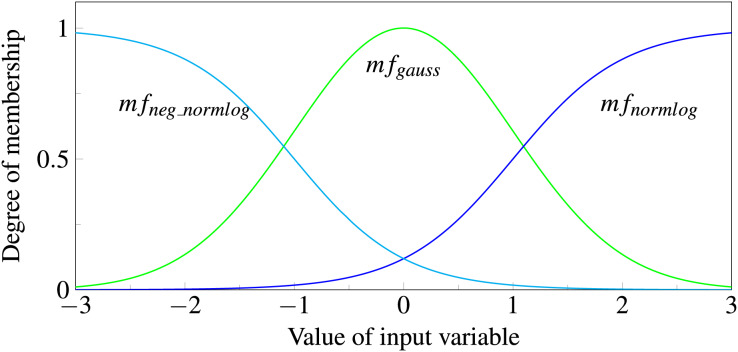
Normlog, Gaussian and negative Normlog act as membership functions, respectively denoted as $ mf_{normlog}$, $ m{f_{neg\_gauss}}$, and $ m{f_{neg\_normlog}}$. For each function the input variable gets a degree of membership, where the maximum value determines whether the variable is treated as “negative”, “zero” or “positive”.

Each input variable is passed to all three functions. The function outputs correspond to the degree of membership of the fuzzy set represented. We consider an input to be a member of “negative” if $m{f_{neg\_normlog}}(x)$ has the highest value, a member of “zero” if it is $m{f_{gauss}}(x)$, and a member of “positive” if it is $m{f_{normlog}}(x)$ (see Fig. 6). If the data was preprocessed with first differences, which is often done for time series, the interpretations of the three classes are decreasing, constant and increasing, respectively. During model training the function parameters, *e.g*., the width $\sigma$ of the Gaussian or the slope $\lambda$ of the Normlog, are adjusted, which changes the range for which the input variables are assigned to the specific classes. During training, the model learns what range of inputs corresponds to which class. For example, a volatile stock can be seen as constant in a broader range than a slow changing stock. Because these parameters are input specific, each input ${x_i}$ has own membership functions with varying parameters $m{f_{neg\_normlog,i}}$ *etc*.

In the second step, *fuzzy inference*, we design rules between input and output variables that the network can follow, like: “IF ${{\bf{x}}_{t,i}} = zero$ AND ${{\bf{x}}_{t,j}} = negative$ THEN ${{\bf{y}}_{t,i}} = negative$”.

Conditions are modeled using the membership functions. For instance, “IF ${{\bf{x}}_{t,i}} = zero$” can be modeled as ${\bf{b}}({{\bf{x}}_{t,i}}) = m{f_{gauss,i}}({{\bf{x}}_{t,i}})$, since the membership function has an output significantly higher than zero if ${{\bf{x}}_{t,i}}$ is close to zero. OR rules are built by using two IF rules, *e.g*., instead of “IF ${{\bf{x}}_{t,i}} = zero$ OR ${{\bf{x}}_{t,j}} = negative$ THEN ${{\bf{y}}_{t,i}} = negative$” use “IF ${{\bf{x}}_{t,i}} = zero$ THEN ${{\bf{y}}_{t,i}} = negative$” and “IF ${{\bf{x}}_{t,j}} = negative$ THEN ${{\bf{y}}_{t,i}} = negative$”. AND rules can be modeled using the product of membership functions, *e.g*., for the rule above: ${\bf{b}}({{\bf{x}}_{t,i}},{{\bf{x}}_{t,j}}) = m{f_{gauss,i}}({{\bf{x}}_{t,i}}) \cdot m{f_{neg\_normlog,j}}({{\bf{x}}_{t,j}})$.

This can be extended to the $m$ inputs with each three membership functions (see Eq. (9)) and rewritten to:

(10) $${{\bf{b}}_k}({\bf{x}}) = \prod\limits_{i = 1}^m {\prod\limits_{l = 0}^2 m } {f_{l,i}}{({{\bf{x}}_i})^{{{\bf{P}}_{k,3i + l}}}}$$

where ${{\bf{b}}_k}$ represents the degree to which the condition for the $k$-th rule is fulfilled and ${{\bf{P}}_{k,3i + l}} = 1$ if the $i$-th input with the $l$-th membership function is part of the rule and ${{\bf{P}}_{k,j}} = 0$ else. For every new rule, a new row is added to the matrix ${\bf{P}}$. In this way, any number $r$ of rules can be created. Since neural networks are not able to perform such computations, we have to rewrite the equation into a sum with activation functions:

(11) $${{\bf{b}}_k}({\bf{x}}) = \prod\limits_{i = 1}^m {\prod\limits_{l = 0}^2 m } {f_{l,i}}{({{\bf{x}}_i})^{{{\bf{P}}_{k,3i + l}}}} = \exp \left( {\sum\limits_{i = 1}^m {\sum\limits_{l = 0}^2 {{{\bf{P}}_{k,3i + l}}} } \ln \left( {m{f_{l,i}}({{\bf{x}}_i})} \right)} \right)$$

After calculating the degrees ${\bf{b}}({\bf{x}})$ to which the rules are fulfilled, we multiply with a matrix ${\bf{C}} \in {\mathbb R}^{3 \times r}$. It models the consequences of the rules by connecting the entries of ${\bf{b}}({\bf{x}})$ to three classes negative, zero and positive:

(12) $${{\bf{x}}_{interpretable}} = {\bf{C}} \cdot softmax({\bf{b}})$$

For each rule $i$, the column $i$ of ${\bf{C}}$ models the respective consequence by setting the connection to either one or zero. For instance, if the consequence of a rule is negative, only the entry in ${\bf{C}}$ that connects ${{\bf{b}}_i}$ and the class that represents negative is one and the entries to the classes zero and positive are zero. During training the entries of ${\bf{C}}$ are adjusted and can be seen as a measure of how much the model trusts each rule given the data. The entries of ${{\bf{x}}_{interpretable}}$ in Eq. (12) show to what degree the forecast is negative, zero and positive.

Finally, the *defuzzification* translates the interpretable output ${{\bf{x}}_{interpretable}}$ back to a numerical value with ${\bf{W}} \in {\mathbb R}^{1 \times 3}$:

(13) $${{\bf{y}}_{t + 1}} = {\bf{W}}{{\bf{x}}_{interpretable}}$$

**Implementation. **For *fuzzification*, we have written a torch.nn.Module for each of the three membership functions defined in Eq. (9) and another one that applies them to any input variable. So for each input variable, the parameters of the three functions are trained independently. The output of the *fuzzification* has one dimension more than the input since each input variable results in three return values.

To initialize a Fuzzy-NN, we first have to define the membership functions and their meaning. With the proposed structure of MemberLayer users can add and replace membership functions to enable new ways of model and data interpretation.


membership_fcts = {
 "negative": NormlogMember(negative=True),
 "zero": GaussianMember(),
 "positive": NormlogMember()}
fuzzification = Fuzzification(
 n_features_input=n_input,
 membership_fcts=membership_fcts)

In *fuzzy inference* the RuleManager class loads the user-defined rules from a json file. They consist of conditions and consequences, the name and position of the input names and the interpretation of the membership functions. The RuleManager automatically creates the corresponding matrices ${\bf{P}}$ and ${\bf{C}}$. The matrix ${\bf{P}}$ is implemented with a convolutional layer $torch.nn.Conv1d$, since the output of the fuzzification step is two dimensional (number of input variables times the number of rules). With these rules we can initialize the torch.nn.Linear layer that performs Eq. (11). It is possible to create an arbitrary number of rules and output classes. So the interpretation of the output is not limited to low, constant and high, but allows adaption to various use cases.


rule_manager = RuleManager(
 path="rules.json",
 rule_matrix_shape=(3m, n_input, 3),
 classification_matrix_shape=(r, 3))
fuzzy_inference = FuzzyInference(
 n_features_input=m,
 n_rules=r,
 n_output_classes=3,
 n_membership_fcts=3,
 rule_matrix=rule_manager.rule_matrix,
 classification_matrix=rule_manager.classification_matrix)

In the *defuzzification*, first the matrix ${\bf{C}}$ is applied so that we get the interpretable output. Afterwards, a softmax function and the matrix ${\bf{W}}$ are applied.


defuzzification = Defuzzification(n_output_classes=3)

Finally, after all layers are initialized, we can create the Fuzzy Neural Network that can be trained like any other PyTorch module:


fuzzy_nn = torch.nn.Sequential(fuzzification, fuzzy_inference,
    defuzzification)

### Visualizations of forecasts and uncertainties

Ensembles are frequently used to quantify uncertainty of neural networks (Lakshminarayanan, Pritzel & Blundell, 2017; Van Schaeybroeck & Vannitsem, 2016). Köpp, Von Mettenheim & Breitner (2014) present an approach using heatmaps to visualize tiny accumulations and paths taken by many models. An implementation is only available in Java (Köpp, Von Mettenheim & Breitner, 2014). We implement this visualization in Python and demonstrate it using a single output variable as an example.

**Theory.** To display the heatmap continuously, we interpolate the values between the discrete time steps in the forecast. Based on these continuous forecasts, we then determine the heat of every pixel in the heatmap.

Let $d \in {\mathbb N}$ be the number of forecasts, $p \in {\mathbb N}$ the number of interpolated values between two time steps and ${y_{res}} \in {\mathbb N}$ the resolution in the y-axis. The number of pixels in the heatmap is therefore ${y_{res}} \times p\tau$. Increasing either $p$ or ${y_{res}}$ smoothens the heatmap, but with computational costs.

We interpolate all forecasts linearly between all time steps:

(14) $$\hat y_{t + j/p}^{[i]} = \left\{ {\matrix{ {{y_T} + {j \over p} \cdot (\hat y_{T + 1}^{[i]} - {y_T})} \hfill & {{\mathrm{if}}\;t = T} \hfill \cr {\hat y_t^{[i]} + {j \over p} \cdot (\hat y_{t + 1}^{[i]} - \hat y_t^{[i]})} \hfill & {{\mathrm{if }}\;t = T + 1, \ldots ,T + \tau } \hfill \cr } } \right. \quad {\mathrm{ for }} \;\;\matrix{ {i = 1, \ldots ,d} \hfill \cr {j = 1, \ldots ,p - 1} \hfill \cr }$$

where $j/p$ defines the intermediate time steps between periods and $\hat y_{t + j/p}^{[i]}$ represents the interpolated value for the $i$-th forecast and time step $t + j/p$.

Next, we calculate a matrix $H \in {\mathbb R}^{{y_{res}} \times (p\tau )}$ where each entry represents the heat of one pixel in the figure. There is a column for each interpolated time step $t = T,T + {1 \over p}, \ldots ,T + {{p\tau } \over p}$. The heat is based on the difference between the value the pixel represents and the forecasted values. Therefore, we assign each row ${H_u}$ in the figure a value $f(u)$ on the same scale as $y$ and the forecasts. We define the bottom pixel row ( $u = 1$) as the minimum of the forecasted values and the last known value ${y_T}$: ${\hat y_{min}} = \min (\{ \hat y_t^{[i]}\;|\;t = T, \ldots ,T + \tau ;\;i = 1, \ldots ,d\} \cup \{ {y_T}\} )$ and the top row ( $u = {y_{res}}$) as the maximum: ${\hat y_{max}} = \max (\{ \hat y_t^{[i]}\;|\;t = T, \ldots ,T + \tau ;\;i = 1, \ldots ,d\} \cup \{ {y_T}\} )$. The values of the rows in between are interpolated linearly: $f(u) = {\hat y_{min}} + {u \over {{y_{res}}}}({\hat y_{max}} - {\hat y_{min}})$.

Each forecast contributes to the total heat of the $t$-th pixel in row $u$ with the difference between the value the pixel represents $f(u)$ and the forecast $\hat y_t^{[i]}$. The difference is weighted by the Gaussian kernel ${k_{Gaussian}}(\delta ) = {\exp ^{ - {{({\delta \over \sigma })}^2}}}$ with $\sigma \in {\mathbb {R}^{+}}$. For each pixel we sum up the heat of all forecasts at the time point:

(15) $${h_{u,v}} = \sum\limits_{i = 1}^d {{{\exp }^{ - {{\left( {{{f(u) - \hat y_v^{[i]}} \over \sigma }} \right)}^2}}}} {\mathrm{ for }}{\eqalign{ {u} & = 1, \ldots ,{y_{res}}\\ {v} & = T,T + {1 \over p}, \ldots ,T + {{p\tau } \over p} \cr}}$$

Since at earlier forecast steps the heat is higher due to denser forecasts, accumulations for less dense regions are hard to identify (Köpp, Von Mettenheim & Breitner, 2014). That is why we normalize each column to have a maximum heat of 1 in each step:

(16) $$h_{u,v}^{norm} = {{{h_{u,v}}} \over {{{\max }_{u = 1, \ldots ,{y_{res}}}}{h_{u,v}}}}{\mathrm{ for }}\;v = T,T + {1 \over p}, \ldots ,T + {{p\tau } \over p}$$

**Implementation. **In order to simplify creating an ensemble, we implemented a torch.nn.Module that takes a base_model and duplicates it, using different start initializations for the weights. With the parameter initializer in Ensemble the user can choose the method for weight initialization. Initializers from torch.nn.init are possible with the default value torch.nn.init.kaiming_uniform_. With n_models we can set the size of the ensemble:


ensemble = Ensemble(model=base_model, n_models=n_models,
    initializer=kaiming_uniform_)

The variable forecasts has to be a torch.Tensor with $d$ forecasts in the rows and a prediction step $\tau$ in each column. The heatmap_forecasts function uses scipy’s interp1d function for interpolation and seaborn to visualize the heatmap (Virtanen et al., 2020; Waskom, 2021). The forecasts in the heatmap are depicted sharper with sigma close to zero and smoother with bigger values. In the function call, we have to set the parameters $d,p$ and ${y_{res}}$ (see ‘Theory’). The number of forecasts is taken from the number of rows of forecasts.


heatmap_forecasts(forecasts, sigma, n_interpolation=p,
    y_resolution=y_res)

### Sensitivity analysis

Due to the various application fields, there is growing interest in the interpretability of neural networks for time series analysis (Rojat et al., 2021). Methods can be divided into *post-hoc* and *ante-hoc* as well as local and global ones (Rojat et al., 2021): While *post-hoc* methods examine the relationship of model input and output after predictions, *ante-hoc* methods are embedded within the model architecture, so that interpretability is included in model training. Examples for the latter are N-BEATs (Oreshkin et al., 2019) which can split the forecast into components, attention layers as applied within Transformer models (Vaswani et al., 2017), and fuzzy neural networks as discussed in ‘Fuzzy neural network’. Local interpretability approaches act on single samples, *e.g*., a single forecast, and global ones over a dataset.

We focus on the *post-hoc* global method of sensitivity analysis which describes how the model inputs influence the output (Dimopoulos, Bourret & Lek, 1995) by calculating partial derivatives of the model output with respect to its inputs. Since this method is model agnostic, it is also used as part of saliency maps in image classification with convolutional neural networks (Simonyan, Vedaldi & Zisserman, 2014), which show important parts of an image related to its classification. For more model agnostic *post-hoc* interpretability tools we refer the reader to Lundberg & Lee (2017) and Gevrey, Dimopoulos & Lek (2003).

The sensitivity analysis function in the prosper_nn package identifies the influences of the input variables on the output of PyTorch neural networks. The packages NeuralSens (Pizarroso, Portela & Muñoz, 2022) and NeuralNetTools (Beck, 2018) provide similar functionalities in R. In comparison to available implementations, our tool is not limited to feed forward networks but can handle recurrent structures as well.

**Theory. **A neural network can be seen as a function $f$ mapping an input vector ${\bf{x}} \in {\mathbb R}^{n}$ to an output $f(x)$. The partial derivative of the output with respect to an input variable ${x_i}$, ${{\partial f(x)} \over {\partial {x_i}}}$, identifies the sensitivity of the output to this variable, *i.e*., how the output will change if the variable is altered. With a positive derivative the output will increase if the variable increases, and will decrease if the variable decreases. This relationship is reversed for negative derivatives. The absolute value of the derivation indicates the size of change to the output. Since these relations can be noisy, we propose to use the mean of an ensemble to have more reliable results (Mehringer et al., 2023).

If we calculate the sensitivities of all input variables for many observations, *e.g*., over time, we can classify the variables according to their influence on the model output:

- Constant: the derivations stay almost the same across the observations.
- Monotonic: the sign of the derivations remains the same across the observations.
- Non-monotonic: the sign of the derivations changes across the observations.
- Unrelated: the derivations are all close to zero.

These categories can be interpreted as follows: For a constant influence, there is a linear dependency between input and output. The direction of change depends on the sign. For monotonic results, the direction of change is fixed but the actual effect size can vary. If the sensitivity analysis shows a non-monotonic relation, the dependencies between input and output are more complex. For the unrelated sensitivity category, the output is not sensitive towards changes in the input variable. Hence, we can discard these variables for the purpose of feature selection.

**Implementation. **The sensitivity analysis function takes the trained ensemble and the data for which the sensitivity analysis is performed. Further, we set the output_neuron as a Tuple. From the tensor returned by the model, the tuple selects the node for which the analysis should be performed. This way, the function can process models with arbitrary output formats as long as they are a torch.Tensor and therefore also RNNs or CNNs.


sensitivity_analysis(model=ensemble, data=data,
    output_neuron=output_neuron)

The function returns the sensitivities of the output_neuron with respect to the input variables across the data observations graphically as a heatmap. Variables are displayed on the y-axis and observations on the x-axis. Negative relations are color coded in blue, positive ones in red. The opacity indicates the strength of the influence. Variables with constant influence reveal roughly the same shade of color over the observations. Monotone relations appear as either red or blue rows while non-monotone ones as rows of mixed color. Light colored rows identify variables with weak relations to the output.

## Case study

In this section, we apply the ECNN, HCNN and CRCNN on the FRED-MD data published by McCracken & Ng (2016). The dataset is publicly available at the website of the Federal Reserve Bank of St. Louis (https://research.stlouisfed.org/econ/mccracken/fred-databases/) and updated monthly. It contains macro-economic indicators. We use the version *2024-07* for this case study.

To benchmark the prosper_nn models, we compare them to the naive (no-change) forecast and nine different RNNs.

To demonstrate how to analyze the forecast uncertainty, we create the uncertainty heatmap for one variable predicted by the ECNN. Then, we investigate the relationship between the ECNN’s one-step forecast and the present values of the other exogenous variables, using the sensitivity analysis.

The code for this case study is available at GitHub (https://github.com/Fraunhofer-IIS/prosper_nn/tree/38279ca2621f3d7de3fe746581e56487a5cf0d01/examples/case_study) and preserved on Zenodo under Beck et al. (2024). All details and configurations to reproduce the study can be found there. More example scripts for the other models can be accessed on the GitHub repository.

**The data.** From the FRED-MD data, we select three groups of variables. For each of these groups we perform the benchmark independently. The groups are “Prices” (19 price-related variables without OILPRICEx), “Output and Income” (16 related variables), and “Consumption and Orders” (10 related variables). The variables that belong to the groups can be found in the Appendix of McCracken & Ng (2016) or in our repository (https://github.com/Fraunhofer-IIS/prosper_nn/blob/63d6dec0b70e0c0ebd7027e69db8eff2f89cda92/examples/case_study/fredmd.py).

Since the time series are not stationary, they are transformed into compound returns by applying the logarithm and first differences: ${\bf{y}}{^\prime _t} = \ln ({{\bf{y}}_t}) - \ln ({{\bf{y}}_{t - 1}})$. We then create rolling origins with 24 months past (past_horizon = 24) and 3 months forecast horizon (forecast_horizon = 3). Depending on where the forecast horizon belongs to, the rolling origins are assigned to the train set (1959-01-01 to 2009-12-31), validation set (2010-01-01 to 2010-12-31) or the test set (2011-01-01 to 2024-06-30). Rolling origins with forecast horizons in two sets are ignored. Each rolling origin is standardized by subtracting the mean and dividing by the standard deviation of the rolling origin for each variable. It follows for the rolling origin with forecast start at ${t_0}$:

${\bf{y}}^{\prime \prime} _t = \displaystyle{{{\bf{y}}^{\prime} _t - mean\left( {{\bf{y}}^{\prime} _{{t_0} - 24}, \ldots ,{\bf{y}}^{\prime } _{{t_0}}} \right)} \over {std\left( {{\bf{y}}^{\prime} _{{t_0} - 24}, \ldots ,{\bf{y}}^{\prime} _{{t_0}}} \right)}}$ for all $t = {t_0} - 24, \ldots ,{t_0}$.

**Initializing the prosper_nn model. **In the following, we describe the ECNN initialization for the “Prices” group. The initialization of the HCNN and CRCNN and for the other data groups is analogous. Although it is possible to forecast multiple target features by a single ECNN, for this case study we restrict ourselves to one target n_features_Y = 1. This means, we train 19 ECNN models in total in order to forecast every variable in the “Prices” group. Since we use 18 exogenous variables in the ”Prices” group plus the past of the target variable as input to the model, we set n_features_U = 19. Then we initialize a single ECNN with a hidden state dimension of twice the size of the input n_state_neurons = 38. Since in the dataset the exogenous variables are unknown for the future, we set future_U = False. Next, we create an ensemble of n_models = 25 members. This allows a detailed visualization of the forecast uncertainty in the heatmap and reliable interpretations in the sensitivity analysis in the following sections.


ecnn = ECNN(
  n_state_neurons=38,
  n_features_Y=1,
  n_features_U=19,
  past_horizon=24,
  forecast_horizon=3,
  future_U=False)
ensemble = Ensemble(model=ecnn, n_models=25)

**Training the prosper_nn model.** During training, we iterate over epochs and batches. The batches are created from the rolling origins described above. We pass features_past, which contains all exogenous variables in the past, plus the the past of the target variable target_past. Additionally, we pass the future target as third argument target_future in order to calculate the loss. From the ensemble output, we extract the mean and split the past horizon so that we keep only the forecasts. They are compared to the future target using the loss function. The training loop itself and the steps per epoch are typical for PyTorch models: First, the gradients in the model are set to zero, second, the prediction is calculated, third, the prediction is used to calculate the loss, fourth, the gradients are calculated by the backward method, and fifth, the optimizer updates the model weights. This way the model is trained by error backpropagation through time.


loss_function = nn.MSELoss()
optimizer = optim.Adam(ensemble.parameters())
for epoch in range(n_epochs):
    for features_past, target_past, target_future in dataloader:
        ensemble.zero_grad()
        ensemble_output = ensemble(features_past, target_past)
        mean = ensemble_output[−1]
        _, forecasts = torch.split(mean, past_horizon)
        loss = loss_function(forecasts, target_future)
        loss.backward()
        optimizer.step()

For HCNN and CRCNN, the training loop is identical except for the minimizing of the loss. Since both models are multivariate, the models predict all variables at once. Therefore, we not only minimize the loss across the three forecast steps, but also across all variables in the corresponding variable group.

**Benchmark models. **We aim to compare the performance of the ECNN, HCNN and CRCNN to other common RNNs. As benchmark RNNs we use the three recurrent cells Elman, GRU and LSTM and we combine each variant with the forecast methods *direct*, *recursive* and *sequence to sequence (s2s)*, so we end up with nine different RNNs. For each, we calculate the context vector ${{\bf{s}}_T}$ with the specified recurrent cell. The forecast is created as follows for the forecast methods:

- *direct*: Affine linear transformation from the context vector to forecast all steps ${\widehat {\bf{y}}_t}$ for $t = T + 1, \ldots ,T + \tau$.
- *recursive*: Use the ${\bf{same}}$ recurrent cell to create states ${{\bf{s}}_t}$ for $t = T + 1, \ldots ,T + \tau$, followed by an affine linear transformation from ${{\bf{s}}_t}$ to ${\widehat {\bf{y}}_t}$ for $t = T + 1, \ldots ,T + \tau$.
- *sequence to sequence (s2s)*: Use a ${\bf{second}}$ recurrent cell of the specified type to create states ${{\bf{s}}_t}$ for $t = T + 1, \ldots ,T + \tau$, followed by an affine linear transformation from ${{\bf{s}}_t}$ to ${\widehat {\bf{y}}_t}$ for $t = T + 1, \ldots ,T + \tau$.

All models are trained like described for the ECNN and with the same hyperparameters. We iterate a maximum of 50 epochs, but stop earlier if the validation loss does not decrease for ten epochs. The validation loss is calculated on all rolling origins of the validation set. Afterwards, we use the model weights with the smallest validation loss. For each variable in each variable group, we trained a separate model.

To have a baseline model to compare to, we also include the naive forecast in our study.

**Figure 7:**
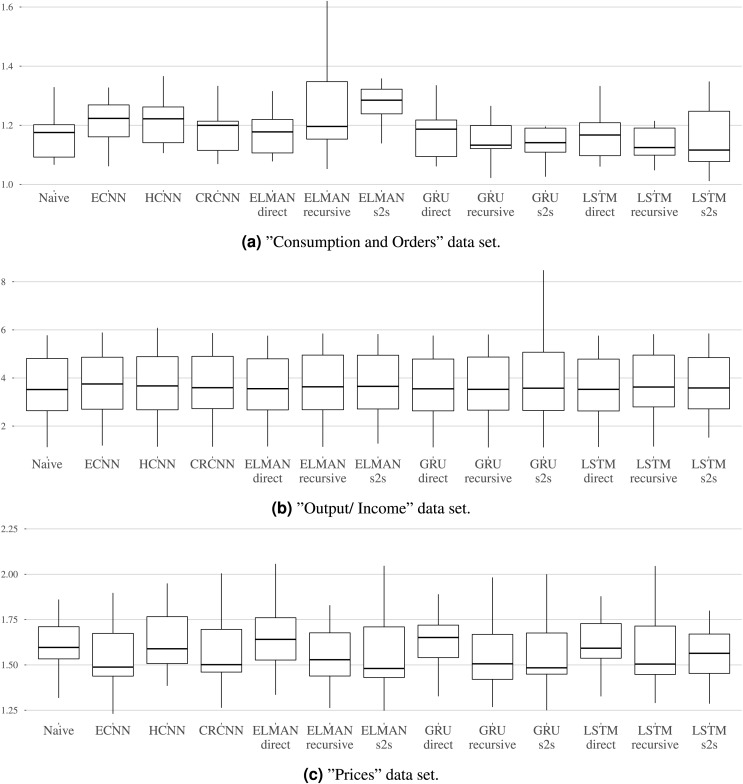
(A–C) The mean MSE over the three horizons is calculated per variable. The distribution of mean MSEs over the variables is shown per model. Each graphic refers to one dataset.

**Benchmark results. **Table 2 shows the MSE over all rolling origins in the test set and over all variables in the groups “Consumption and Orders”, “Output and Income”, and “Prices” for up to three forecast steps. Additionally, Figs. 7A–7C show the distribution of MSEs per dataset and the different models.

**Table 2:** Average MSE over the variables per horizon, dataset, and model. The best ranking method is marked bold and the second best is underlined.

	Consumption/orders			Output/income			Prices		
Model	$h = 1$	$h = 2$	$h = 3$	$h = 1$	$h = 2$	$h = 3$	$h = 1$	$h = 2$	$h = 3$
Naive	1.18	**1.16**	**1.16**	**2.44**	**4.42**	4.70	1.47	1.68	1.74
ECNN	1.07	1.27	1.30	2.48	4.56	4.78	1.30	1.64	1.74
HCNN	1.25	1.21	1.20	2.52	4.54	4.77	1.39	1.73	1.84
CRCNN	1.19	1.17	1.19	2.50	4.54	4.70	1.34	1.65	1.72
ELMAN (direct)	1.16	1.20	1.17	2.45	4.43	4.72	1.48	1.71	1.75
ELMAN (recursive)	1.14	1.31	1.34	2.48	4.53	4.74	1.32	1.65	1.75
ELMAN (s2s)	1.10	1.35	1.38	2.49	4.55	4.75	**1.29**	1.64	1.74
GRU (direct)	1.18	1.17	1.17	**2.44**	**4.42**	4.69	1.46	1.72	1.76
GRU (recursive)	1.05	1.25	1.25	2.49	4.50	4.70	1.34	**1.62**	**1.71**
GRU (s2s)	1.05	1.24	1.25	2.49	4.52	4.70	1.32	1.63	**1.71**
LSTM (direct)	1.17	**1.16**	**1.16**	**2.44**	**4.42**	**4.68**	1.47	1.69	1.75
LSTM (recursive)	**1.01**	1.23	1.23	2.53	4.56	4.75	1.38	1.65	1.73
LSTM (s2s)	1.03	1.23	1.22	2.52	4.53	4.73	1.36	1.63	1.72

For the “Consumption and Orders” variable group, the naive and the LSTM with direct forecast perform best for the individual forecasting steps while the recursive and sequence-to-sequence variant of the GRU and recursive variant of the LSTM seem to work best overall. The prosper_nn models are not able to keep up with this but still give competitive results, especially when looking at the individual forecasting steps.

For the “Output and Income” dataset, the performance differences between the models in the benchmark are very small. No method is really able to outperform the naive forecast. This suggests that the variables in this group are difficult to forecast for recurrent neural networks in general. The prosper_nn models perform very similar to the other RNNs in the benchmark.

For the “Prices” group, the ECNN and the CRCNN belong to the models with the lowest MSE median over all forecast steps (see Fig. 7C). The median of the ELMAN with sequence-to-sequence is smaller than both, but ECNN and CRCNN have less variance in their errors. Also, the ECNN performs very well on the individual forecasting steps (see Table 2)

Overall in this case study, the prosper_nn have proven to be competitive with other RNN variants. Depending on the data and the evaluation focus, they are able to outperform other methods.

Although the benchmark includes 45 variables with over 150 rolling origins, due to the limited dataset size, this benchmark can only serve as illustrative example and we leave more extensive benchmarks to future work.

**Forecast uncertainty of ECNN ensemble.** Next, we are interested in the ensemble forecast uncertainty of the ECNN. As an example we take the variable DNDGRG3M086SBEA as the target, which is the personal consumption expenditures (PCE) of nondurable goods. We take all the forecast scenarios of the ensemble members, but ignore the last entry of the forecasts tensor, which contains the mean forecast of the scenarios. In the second dimension (the time dimension), we only keep the forecast horizon. Since we have only one variable in the last dimension, we select it directly. To see the forecast in the original scale we post-process the data, *i.e*., we reverse the standardization and calculate the forecasts back from the compound returns. We then calculate the uncertainty heatmap from the forecasts. The function takes the start_point as an optional parameter. This is the last known value of the target variable and if passed, it is also shown in the heatmap.


rolling_origin_start_date = pd.Period("2011-01-01", freq="M")
features_past, target_past, _ = fredmd_test.get_one_rolling_origin(
    rolling_origin_start_date
    )
ensemble.eval()
with torch.no_grad():
   forecasts = ensemble(features_past, target_past)
forecasts = forecasts[:−1, past_horizon:, 0]
forecasts = fredmd_test.rescale(forecasts, rolling_origin_start_date)
forecasts = fredmd_test.postprocess(forecasts,
    rolling_origin_start_date)
start_point = torch.tensor(
    fredmd_test.original_data.loc[rolling_origin_start_date - 1,
    fredmd_test.target]
    )
heatmap_forecasts(forecasts[..., 0], start_point=start_point)

**Figure 8:**
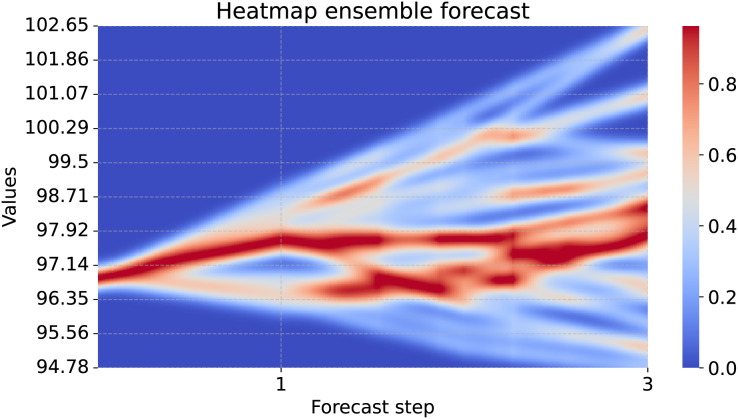
Uncertainty heatmap of a 25 member ensemble. The x-axis shows three forecast steps and the y-axis shows the scale of the forecasted values. According to the model, it is unlikely that the true value will be in dark blue regions. For brighter blues and darkening red regions the model predicts higher probabilities according to the color bar on the right side.

In Fig. 8 we see the forecast of the example variable (PCE of nondurable goods). It shows the advantage of the uncertainty heatmap over just plotting the ensemble mean or individual lines of the forecasts: We can identify areas in which the forecasts accumulate. In the one-step forecast, most forecasts are close to each other. For further steps, the forecast members diverge and the uncertainty increases. Using the uncertainty heatmap, we still are able to identify areas that are more common than others. For example, in the second step there are two regions with high probability, but in between there is an area of low probability. Therefore, a decision can be taken according to one of the two scenarios, instead of a mixture of both as the mean might suggest.

**Sensitivity analysis. **Next, we explore which relationships the model has learned. We again take the PCE of nondurable goods as the example target variable. We pass the ensemble and the data to the sensitivity_analysis function. In this case study, we are interested in how changed values at the forecast origin would influence the one-step ahead forecast. For the output_neuron follows: The first dimension (−1) selects the mean output of the ensemble members, past_horizon + 1 the one-step forecast, 0 the index of the (only) sample in the batch and 0 the index of the variable we want to observe. To improve readability, we restrict the plot on the influences at the forecast origin sensitivity[:, −1]. Then we plot the restricted_sensitivity_matrix.


all_ros_features_past, all_ros_targets_past, _ = (fredmd_test
    .get_all_rolling_origins())
all_ros_features_past = (all_ros_features_past.transpose(1, 0)
    .unsqueeze(2))
all_ros_targets_past = (all_ros_targets_past.transpose(1, 0)
    .unsqueeze(2))
sensitivity = sensitivity_analysis(
    ensemble,
    *(all_ros_features_past, all_ros_targets_past),
    output_neuron=(−1, past_horizon + 1, 0, 0),
    batchsize=1
)
restricted_sensitivity_matrix = sensitivity[:, −1].squeeze(1)
seaborn.heatmap(restricted_sensitivity_matrix.T)

**Figure 9:**
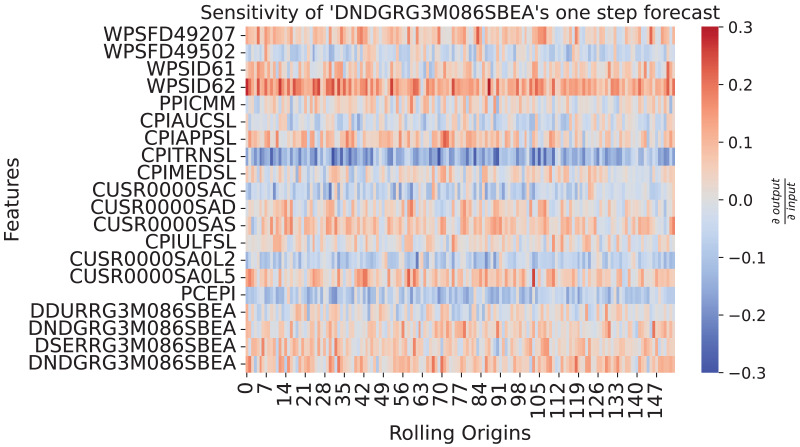
Sensitivity heatmap of the ensemble for one-step forecast of DNDGRG3M086SBEA. The y-axis shows the input features plus the influence of DNDGRG3M086SBEA *via* the error correction mechanism in the last row. The x-axis goes over all rolling origins of the test set. The color shows the sensitivity of the model with respect to the input, where red represents a positive relationship and blue a negative one. As depicted by the colorbar on the right side, strong colors indicate a strong sensitivity.

Figure 9 shows how the past values influence the one-step forecasts of PCE: Nondurable goods. The sensitivity shows a continuous strong negative relationship to CPITRNSL, which is the consumer price index of transportation. This means, lower values for CPITRNSL at the forecast origin leads to the model producing a higher forecast for PCE: Nondurable goods. This aligns with the interpretation that lower costs for transportation can increase consumption of nondurable goods since prices are lower.

## Discussion

To make the package usable for many researchers and practitioners we chose Python as a programming language and PyTorch as the framework for implementation. Both are widely used and system independent. Additionally, PyTorch allows users to easily train the models on GPUs. A suitable environment can be created with the dependencies listed in the requirements (https://github.com/Fraunhofer-IIS/prosper_nn/blob/main/requirements.txt).

For ease of use we set sensible default values for parameters where possible, while keeping many options to tune them. Typing and explanations for each parameter can be found in the API reference. Utility functions like the sensitivity analysis and the uncertainty heatmap are demonstrated there in a short code example. For the models, the documentation includes a short introduction to the model theory, the formulas the implementation is based on, references and a short example on how to train the model. The package documentation (https://fraunhofer-iis.github.io/prosper_nn/) also contains longer tutorials in the form of Jupyter notebooks (Kluyver et al., 2016). For code quality, we set typings in the code and automatically check the code with flake8 as a GitHub action for different Python versions.

The implementation of the recurrent model architectures (ECNN, HCNN and CRCNN) stays close to the one of the RNN in PyTorch to make the code reusable. We implement recurrent cells that model one time step and then stack cells in order to create an overall model. Each cell and the models are implemented as PyTorch modules. Since CRCNNs use HCNNs internally, there is no extra CRCNN cell. For fuzzy neural networks, we applied a modular approach as well. This way, individual modules can be changed or new ones can be added by users.

Overall, with its modular design the package is meant to make it easy to apply the mentioned models, even if not familiar with the theory or the implementation, while also providing the option to adapt the code or add further modules. This can be done by cloning the package from GitHub. On GitHub, contributions can be submitted with a pull request and issues or discussions can be opened.

The software is already in use for steel price and demand forecasting in industry, for sensitivity analysis (Mehringer et al., 2023) and in student theses. The models in prosper_nn cover many forecasting use case conditions. ECNN are suitable for almost all tasks a typical RNN could solve. HCNN and CRCNN are more suited for multivariate forecasting like in macroeconomic or weather forecasting. For use cases where much explainability is needed, *e.g*., financial forecasting, fuzzy neural networks are suitable.

The sensitivity analysis is not restricted to forecasting, and can be applied to any neural network, even beyond the ones implemented in the prosper_nn package, to understand the influences of the input on the predictions. To visualize uncertainty the forecast heatmap can be used wherever uncertainty is determined with different forecast paths, *e.g*., Monte Carlo dropout, ensemble or Bayesian inference. This makes it widely usable and not restricted to neural networks.

Due to their sequential nature, there is an inherent similarity of time series and language data. While RNNs solve a regression problem for numerical time series data, they can also predict the next word for natural language, which can be seen as a classification problem due to the discrete output space (Graves, 2014). Nevertheless, both data types can be modelled by similar architectures, which enables analogous concepts for language to those discussed for time series. Teacher forcing for example, uses the target word as additional input to the hidden state during training. Sensitivity analysis can also be performed by computing partial derivates of the output classes to the input tokens (Gholizadeh & Zhou, 2021). For a discussion on uncertainty estimation for natural language processing, we refer the interested reader to Xiao & Wang (2019).

We hope that prosper_nn can lie the foundation for future work on the introduced forecasting methods. Especially we would be interested in (i) more extensive benchmarks in contrast to mostly hard to replicate case studies that constitute most of the existing research, (ii) the integration of the ECNN and HCNN cells in other architectures like for example DeepAR, where currently LSTM cells are used, and (iii) the performance of the introduced models if they are trained in a global fashion, *i.e*., over many time series simultaneously. This is the predominant training scheme for most of the currently successful neural networks in forecasting but has not been used for the models in prosper_nn.

## Conclusion

We introduced the PyTorch package prosper_nn which is the first publicly available implementation of ECNNs, HCNNs, CRCNNs and fuzzy neural networks. ECNNs, HCNNs and CRCNNs are recurrent model architectures suited for time series forecasting that use prediction errors to improve performance. ECNNs extend on classical RNNs by correcting the hidden state by the prediction error in order to enhance training. They are mostly suited for short-term forecasting. HCNNs model multivariate time series instead of using exogenous variables as inputs. As a result, they are also suited for long-term forecasting. CRCNNs combine the modeling of past information influencing the future *via* a forward HCNN with the modeling of future information influencing the past *via* a backward HCNN. Therefore, they are suited for use cases where we expect future values to have an influence on current or past values. Fuzzy neural networks are a way to make neural networks interpretable and to easily include expert know-how by using member functions to translate between semantic and numerical expressions. They can be used for regression and classification. Additionally, prosper_nn includes a heatmap function for visualizing the spread of an ensemble forecast and a sensitivity analysis function for analyzing (temporal) dependencies between the model’s input and output. We published the package together with thorough documentation, tutorials, and the code of the case study open source at GitHub: https://github.com/Fraunhofer-IIS/prosper_nn. Our central contribution with prosper_nn is to make all of these methods for the first time available in open source code and accordingly allow for a rigorous evaluation and further development.

In the case study, we showed that the implemented models are easy to apply and are competitive against Elman, GRU and LSTM RNNs as well as the naive forecast across three groups of the FRED-MD dataset. Further, we demonstrated the usage and interpretation of the heatmap and sensitivity analysis for forecasts of the FRED-MD dataset.

We hope this publication leads to further development and practical evaluation of the aforementioned model architectures. Hereby we want to encourage researchers and practitioners to make pull requests and get in contact with us *via* the GitHub repository.

## Additional Information and Declarations

### Data Availability

The following information was supplied regarding data availability: The FRED-MD data set is available at: - https://research.stlouisfed.org/econ/mccracken/fred-databases. Version 2024-07 was used in this article. - https://doi.org/10.1080/07350015.2015.1086655. The prosper_nn code including case study is available at GitHub and Zenodo: - https://github.com/Fraunhofer-IIS/prosper_nn - Beck, N., Schemm, J., Ehrig, C., Frechen, H., Sonnleitner, B., & Neumann, U. (2024). Prosper_nn (0.3.0). Zenodo. https://doi.org/10.5281/zenodo.10906850.

## References

[ref-26] Abadi M, Barham P, Chen J, Chen Z, Davis A, Dean J, Devin M, Ghemawat S, Irving G, Isard M, Kudlur M, Levenberg J, Monga R, Moore S, Murray DG, Steiner B, Tucker P, Vasudevan V, Warden P, Wicke M, Yu Y, Zheng X (2016). Tensorflow: a system for large-scale machine learning.

[ref-1] Abdel-Karim BM, Benlian A, Hinz O (2021). The predictive value of data from virtual investment communities. Machine Learning and Knowledge Extraction.

[ref-2] Beck MW (2018). Neuralnettools: visualization and analysis tools for neural networks. Journal of Statistical Software.

[ref-3] Beck N, Schemm J, Ehrig C, Frechen H, Sonnleitner B, Neumann U (2024). Prosper_nn.

[ref-4] Benidis K, Rangapuram SS, Flunkert V, Wang Y, Maddix D, Turkmen C, Gasthaus J, Bohlke-Schneider M, Salinas D, Stella L, Aubet FX, Callot L, Januschowski T (2023). Deep learning for time series forecasting: tutorial and literature survey. ACM Computing Surveys.

[ref-5] Bjarnle J, Holmström E (2015). Implementation and evaluation of historical consistent neural networks using parallel computing.

[ref-7] Cho K, van Merrienboer B, Gulcehre C, Bahdanau D, Bougares F, Schwenk H, Bengio Y (2014). Learning phrase representations using RNN encoder-decoder for statistical machine translation.

[ref-8] Dimopoulos Y, Bourret P, Lek S (1995). Use of some sensitivity criteria for choosing networks with good generalization ability. Neural Processing Letters.

[ref-6] Erdem Ç, Zimmermann HG (2001). Segmental duration control with asymmetric causal retro-causal neural networks.

[ref-9] Gevrey M, Dimopoulos I, Lek S (2003). Review and comparison of methods to study the contribution of variables in artificial neural network models. Ecological Modelling.

[ref-10] Gholizadeh S, Zhou N (2021). Model explainability in deep learning based natural language processing.

[ref-11] Goodfellow I, Courville A, Bengio Y (2016). Deep learning. Adaptive Computation and Machine Learning.

[ref-12] Granger CWJ (1969). Investigating causal relations by econometric models and cross-spectral methods. Econometrica.

[ref-13] Graves A (2014). Generating sequences with recurrent neural networks.

[ref-14] Hewamalage H, Bergmeir C, Bandara K (2021). Recurrent neural networks for time series forecasting: current status and future directions. International Journal of Forecasting.

[ref-15] Hochreiter S, Schmidhuber J (1997). Long short-term memory. Neural Computation.

[ref-16] Hoffmann D (2015). *Langfristige Kapitalmarktsimulationen: Eine empirische Untersuchung der Eignung von Historically Consistent Neural Networks zur langfristigen Kapitalmarktsimulation im Kontext der Alterssicherung: Zugl.: Bremen, Univ., Diss., 2014*, volume 439 of *Schriftenreihe innovative betriebswirtschaftliche Forschung und Praxis*. Kovač, Hamburg.

[ref-17] Jaeger H (2001). The “echo state” approach to analysing and training recurrent neural networks-with an erratum note. Bonn, Germany: German National Research Center for Information Technology GMD Technical Report.

[ref-18] Kluyver T, Ragan-Kelley B, Pérez F, Granger B, Bussonnier M, Frederic J, Kelley K, Hamrick J, Grout J, Corlay S, Ivanov P, Avila D, Abdalla S, Willing C, Jupyter development team (2016). Jupyter notebooks–a publishing format for reproducible computational workflows. Positioning and Power in Academic Publishing: Players, Agents and Agendas.

[ref-19] Köpp C, Von Mettenheim HJ, Breitner MH (2014). Decision analytics with heatmap visualization for multi-step ensemble data. Business & Information Systems Engineering.

[ref-20] Laje R, Buonomano DV (2013). Robust timing and motor patterns by taming chaos in recurrent neural networks. Nature Neuroscience.

[ref-21] Lakshminarayanan B, Pritzel A, Blundell C (2017). Simple and scalable predictive uncertainty estimation using deep ensembles. Advances in Neural Information Processing Systems 30.

[ref-22] Lenhard G, Maringer D (2022). State-ANFIS: a generalized regime-switching model for financial modeling.

[ref-23] Lundberg SM, Lee SI (2017). A unified approach to interpreting model predictions. Advances in Neural Information Processing Systems 30.

[ref-24] Makridakis S, Hibon M (2000). The m3-competition: results, conclusions and implications. International Journal of Forecasting.

[ref-25] Makridakis S, Spiliotis E, Assimakopoulos V (2018). The m4 competition: results, findings, conclusion and way forward. International Journal of Forecasting.

[ref-27] McCracken MW, Ng S (2016). FRED-MD: a monthly database for macroeconomic research. Journal of Business & Economic Statistics.

[ref-28] Mehringer J, Frechen H, Beck N, Sukowski F, Stocker T, Freier D, Schäfer F (2023). Cast control: AI-based explanations of casting defects linking process and quality inspection data.

[ref-29] Mvubu M, Kabuga E, Plitz C, Bah B, Becker R, Zimmermann HG (2020). On error correction neural networks for economic forecasting.

[ref-30] Oreshkin B, Carpov D, Chapados N, Bengio Y (2019). N-beats: neural basis expansion analysis for interpretable time series forecasting.

[ref-31] Paszke A, Gross S, Massa F, Lerer A, Bradbury J, Chanan G, Killeen T, Lin Z, Gimelshein N, Antiga L, Desmaison A, Kopf A, Yang E, DeVito Z, Raison M, Tejani A, Chilamkurthy S, Steiner B, Fang L, Bai J, Chintala S, Wallach H, Larochelle H, Beygelzimer A, d’Alché-Buc F, Fox E, Garnett R (2019). Pytorch: an imperative style, high-performance deep learning library. Advances in Neural Information Processing Systems 32.

[ref-32] Pizarroso J, Portela J, Muñoz A (2022). Neuralsens: sensitivity analysis of neural networks. Journal of Statistical Software.

[ref-33] Rasul K, Ashok A, Williams AR, Ghonia H, Bhagwatkar R, Khorasani A, Bayazi MJD, Adamopoulos G, Riachi R, Hassen N, Biloš M, Garg S, Schneider A, Chapados N, Drouin A, Zantedeschi V, Nevmyvaka Y, Rish I (2023). Lag-llama: towards foundation models for probabilistic time series forecasting.

[ref-34] Rockefeller R, Bah B, Marivate V, Zimmermann HG (2022). Improving the predictive power of historical consistent neural networks.

[ref-35] Rockefeller R, Bah B, Zimmermann HG, Marivate V (2023). Wind power prediction with hcnns for turbines.

[ref-36] Rojat T, Puget R, Filliat D, Del Ser J, Gelin R, Díaz-Rodríguez N (2021). Explainable artificial intelligence (XAI) on timeseries data: a survey.

[ref-37] Rumelhart DE, Hinton GE, Williams RJ (1986). Learning representations by back-propagating errors. Nature.

[ref-38] Salinas D, Flunkert V, Gasthaus J, Januschowski T (2020). Deepar: probabilistic forecasting with autoregressive recurrent networks. International Journal of Forecasting.

[ref-39] Schuster M, Paliwal KK (1997). Bidirectional recurrent neural networks. IEEE Transactions on Signal Processing.

[ref-40] Siekmann S, Neuneier R, Zimmermann HG, Kruse R, Zadeh LA, Kacprzyk J (1999). Neuro fuzzy systems for data analysis. Computing with Words in Information/Intelligent Systems 2: Applications.

[ref-41] Simonyan K, Vedaldi A, Zisserman A (2014). Deep inside convolutional networks: visualising image classification models and saliency maps.

[ref-42] Smyl S (2020). A hybrid method of exponential smoothing and recurrent neural networks for time series forecasting. International Journal of Forecasting.

[ref-43] Stock JH, Watson MW (2001). Vector autoregressions. Journal of Economic Perspectives.

[ref-44] Sussillo D, Abbott LF (2009). Generating coherent patterns of activity from chaotic neural networks. Neuron.

[ref-45] Tokic M, von Beuningen A, Tietz C, Zimmermann HG (2020). Handling missing data in recurrent neural networks for air quality forecasting.

[ref-46] Van Schaeybroeck B, Vannitsem S (2016). A probabilistic approach to forecast the uncertainty with ensemble spread. Monthly Weather Review.

[ref-47] Vaswani A, Shazeer N, Parmar N, Uszkoreit J, Jones L, Gomez AN, Kaiser Ł, Polosukhin I (2017). Attention is all you need. Advances in Neural Information Processing Systems 30.

[ref-48] Veronesi P (2000). How does information quality affect stock returns?. The Journal of Finance.

[ref-49] Virtanen P, Gommers R, Oliphant TE, Haberland M, Reddy T, Cournapeau D, Burovski E, Peterson P, Weckesser W, Bright J, van der Walt SJ, Brett M, Wilson J, Millman KJ, Mayorov N, Nelson ARJ, Jones E, Kern R, Larson E, Carey CJ, Polat İ, Feng Y, Moore EW, VanderPlas J, Laxalde D, Perktold J, Cimrman R, Henriksen I, Quintero EA, Harris CR, Archibald AM, Ribeiro AH, Pedregosa F, van Mulbregt P (2020). Scipy 1.0: fundamental algorithms for scientific computing in Python. Nature Methods.

[ref-50] Von Mettenheim HJ, Dunis C (2014). Trading decision support with historically consistent neural networks. Computational Intelligence Techniques for Trading and Investment, Routledge Advances in Experimental and Computable Economics.

[ref-51] Von Mettenheim HJ, Breitner MH, Jayne C, Yue S, Iliadis L (2012). Forecasting and trading the high-low range of stocks and ETFs with neural networks. Engineering Applications of Neural Networks.

[ref-52] Von Mettenheim HJ, Breitner MH, Helber S, Breitner MH, Rösch D, Schön C, Von der Schulenburg JM, Sibbertsen P, Steinbach M, Weber S, Wolter A (2014). Forecasting daily highs and lows of liquid assets with neural networks. Operations Research Proceedings 2012, GOR (Gesellschaft für Operations Research e.V.).

[ref-53] Walia N, Singh H, Sharma A (2015). ANFIS: adaptive neuro-fuzzy inference system-a survey. International Journal of Computer Applications.

[ref-54] Waskom M (2021). seaborn: statistical data visualization. Journal of Open Source Software.

[ref-55] Williams RJ, Zipser D (1989). A learning algorithm for continually running fully recurrent neural networks. Neural Computation.

[ref-56] Xiao Y, Wang WY (2019). Quantifying uncertainties in natural language processing tasks. Proceedings of the AAAI Conference on Artificial Intelligence.

[ref-57] Zimmermann HG, Grothmann R, Tietz C, Klatte D (2012). Forecasting market prices with causal-retro-causal neural networks. Operations Research Proceedings 2011.

[ref-58] Zimmermann HG, Grothmann R, Tietz C (2016). A new view on economics with recurrent neural networks. Handbook on Computational Intelligence.

[ref-59] Zimmermann HG, Grothmann R, Von Mettenheim HJ, Rausch P (2013). Planning purchase decisions with advanced neural networks. Business Intelligence and Performance Management, Advanced Information and Knowledge Processing.

[ref-60] Zimmermann HG, Grothmann R, Tietz C, Von Jouanne-Diedrich H, Hu B (2011). Market modeling, forecasting and risk analysis with historical consistent neural networks. Operations Research Proceedings 2010.

[ref-61] Zimmermann HG, Neuneier R, Grothmann R, Leung KS, Chan LW, Meng H (2000). Modeling of the German yield curve by error correction neural networks. Intelligent Data Engineering and Automated Learning—IDEAL 2000. Data Mining, Financial Engineering, and Intelligent Agents.

[ref-62] Zimmermann HG, Neuneier R, Grothmann R, Dietterich T, Becker S, Ghahramani Z (2001a). Active portfolio-management based on error correction neural networks. Advances in Neural Information Processing Systems.

[ref-63] Zimmermann HG, Neuneier R, Grothmann R, Dorffner G, Bischof H, Hornik K (2001b). Multi-agent fx-market modeling based on cognitive systems. Artificial Neural Networks—ICANN 2001.

[ref-64] Zimmermann HG, Tietz C, Grothmann R, Montavon G, Orr GB, Müller KR (2012). Forecasting with recurrent neural networks: 12 tricks. Neural Networks: Tricks of the Trade, volume 7700 of Lecture Notes in Computer Science.

[ref-65] Zimmermann HG, Tietz C, Grothmann R, Georgieva P (2013). Historical consistent neural networks: new perspectives on market modeling, forecasting and risk analysis. Advances in Intelligent Signal Processing and Data Mining, volume 410 of Studies in Computational Intelligence.

[ref-66] Zimmermann HG, Tietz C, Grothmann R, Runkler T (2012). Recurrent neural networks for industrial procurement decisions. KI—Künstliche Intelligenz.

